# Treadmill training protects valproic acid-induced autistic features via cerebellar AMPK/PPAR-γ dependent pathway and improves mitochondrial activity in mice

**DOI:** 10.1038/s41598-025-09089-6

**Published:** 2025-07-12

**Authors:** Anwaar M. Shaban, Omnia Ameen, Marwa Omar, Sara A. El Derbaly, Hend R. Omara, Asmaa I. Bayomi, Asmaa A. Abdel Latif, Zainab Ibrahim Elakabawy, Suzan A. Khodir

**Affiliations:** 1 Medical Physiology Department, Faculty of Medicine, Menoufia University, Menoufia, Egypt; 2 Medical Physiology Department, Menoufia National University, Menoufia, Egypt; 3 Anatomy and Embryology Department, Faculty of Medicine, Menoufia University, Menoufia, Egypt; 4 Anatomy and Embryology Department, Menoufia National University, Menoufia, Egypt; 5 Medical Biochemistry and Molecular Biology Department, Faculty of Medicine, Menoufia University, Menoufia, Egypt; 6 Department of Biochemistry and Molecular Biology, Faculty of Medicine, Ibn Sina University for Medical Science, Amman, 16197 Jordan; 7 Psychiatry Department, Faculty of Medicine, Menoufia University, Menoufia, Egypt; 8 Zoology Department, Faculty of Science, Menoufia University, Menoufia, Egypt; 9 Public Health and Community Medicine Department, Faculty of Medicine, Menoufia University, Menoufia, Egypt; 10 Pathology Department, Faculty of Medicine, Menoufia University, Menoufia, Egypt

**Keywords:** Autism, Training, AMPK, Irisin, PPAR-γ, Metrnl, Biochemistry, Genetics, Molecular biology, Neuroscience, Physiology, Biomarkers, Medical research, Molecular medicine, Neurology

## Abstract

Autism spectrum disorder (ASD) is a neurodevelopmental disorder associated with impaired sociality and stereotypic behavior. Endurance training could modulate mitochondrial dysfunction sharing in the pathophysiology of ASD. We investigated the neuroprotective effects of training on VPA-induced ASD in mice. Forty mice were divided into control, Training, VPA, and VPA + Training groups. Mice were subjected to neurobehavioral tests. Assessment of the protein content of serum CRP, irisin, meteorin-like protein (metrnl), cerebellar inflammatory markers, serotonin, and BDNF was done by ELISA. MDA and catalase were also investigated using a colorimetric technique. Cerebellar citrate synthase (CS) enzyme activity was also measured. Cerebellar AMPK, PPAR-ɣ, and metrnl gene expressions were assessed via RT-PCR. Cerebellar immunohistochemical studies of GFAP, Bax, and PPAR-γ markers were conducted. Statistical methods were used in the data analysis, including one-way ANOVA, and t-tests. The VPA group showed significant impairments in social interaction, and cognition in neurobehavioral tests (*P* = 0.000). A significant increase of CRP, MDA, and inflammatory markers associated with a significant reduction in irisin, metrnl, catalase, CS, serotonin, and BDNF (*P* = 0.000) was noticed. Besides, cerebellar AMPK and PPAR-γ gene expressions were down-regulated. Significant cerebellar degenerative changes were also observed (*P* = 0.000). Training dramatically reversed VPA-induced neurobehavioral, biochemical, and cerebellar degenerative changes. Endurance training has anti-inflammatory, anti-apoptotic, and antioxidant properties. Adipo-myokines release, enhanced mitochondrial activity, and activation of AMPK and PPAR-γ pathways could be involved mechanisms. Training programs are a promising strategy for addressing the social and neurobehavioral impairments linked to ASD, according to the muscle-brain interplay.

## Introduction

The neurological condition known as autism spectrum disorder (ASD) can cause a variety of symptoms, including anxiety, disinterest, stereotyped behavior, and difficulties with language and social relationships. ASD puts a costly burden on society and caregivers^1^. Besides, more than 87% of patients with ASD may have motor comorbidities^2^. The global average estimate is that one child out of every 100 has ASD. It is commonly recognized that the likelihood of having ASD is four times higher in men than in women^3^.

ASD is a complicated disorder that involves interactions between genetic, epigenetic, and environmental factors. Environmental factors include pregnancy-related microbiota, environmental contaminants, and pharmacological exposure such as valproic acid (VPA)^4^. VPA is a frequently used medication to treat epilepsy. However, it is an epigenetic modulator and histone deacetylase inhibitor that can change gene activity, induce neuroinflammation, damage DNA, and disrupt mitochondrial energy metabolism by generating oxidative stress^5^. VPA consumption at the fourteenth postnatal day (PND 14) promotes cerebellar apoptosis and neurodevelopmental regressions, both of which lead to behavioral retardations^6^.

Reactive oxygen species (ROS) are produced in excess because of oxidative stress events that occur during mitochondrial activity and are usually scavenged by antioxidants like the catalase enzyme. Mitochondrial dysfunction (MtD) is caused by the scavenger’s enhanced production of ROS. MtD results in more oxidative stress^7^. The frontal brain and cerebellar tissues of autistic mice have aberrant mitochondrial DNA, citrate synthetase, and mitochondrial electron transport chain complexes because of MtD^8^. Mitochondrial citrate synthase (CS) is one of the special markers of the mitochondrial matrix, and its amount and activity depend on the number of mitochondria^9^. The pathogenesis of ASD was more closely associated with neuroinflammation, oxidative stress, MtD, and gliosis^10^.

Peroxisome proliferator-activated receptors (PPARs), transcription factors that are ligand-regulated, belong to the nuclear receptor superfamily. PPAR-γ is the most common PPAR subtype in the central nervous system. The regulation of metabolism, cell division, neuroprotection against oxidative stress, and inflammation, and apoptosis are all influenced by PPAR-γ^11^. Reduced PPAR-γ plays a role in the pathophysiology of ASD^12^.

AMPK keeps the energy balance by promoting the oxidation of fatty acids and the absorption of glucose. It has been found that glucose deprivation reduces hippocampal neuro-apoptosis after prolonged and continuous AMPK activation^13^. In addition, AMPK is crucial for brain neuroinflammatory disorders^14^. AMPK upregulates many antioxidant genes involved in defense against oxidative damage connected to ASD^15^.

Pharmaceutical therapies do not address the fundamental symptoms of ASD. Some medications just try to reduce the co-occurring symptoms associated with ASD^16^. Over the last few decades, research has looked at the connection between training and cognition in various mental illnesses^17^. Two novel adipo-myokines; irisin, and metrnl, are selectively expressed in response to exercise. Reports indicate that metrnl contributes to the stimulation of glial cell proliferation and axonal extension. It could be a novel marker for some neurological disorders, such as ASD^18^. Irisin, a consequence of protein 5 cleavage, has been implicated in the anxiolytic and anti-depressive effects of exercise. Additionally, irisin modifies the catecholaminergic system in adult male rats by changing adrenergic and dopaminergic receptors. Adipose tissue browning, which increases glucose tolerance, decreases inflammation, and stimulates thermogenesis, may also be facilitated by exercise. Prior research has demonstrated that irisin contributes to the etiopathogenesis of ASD^19^.

Training for a long time increases metabolic demands, cerebral blood flow, and has a strong antioxidant effect with increased availability of brain-derived neurotrophic factor (BDNF) that promotes brain flexibility^20^.

Exercise increases PPAR-γ gene expression level. PPAR-γ activates AMPK via phosphorylation^21^. AMPK activation induces mitochondrial biosynthesis. AMPK also increases the expression of antioxidant enzymes, which reduces ROS generation. Furthermore, AMPK may increase antioxidant responses by activating SIRT1-dependent deacetylation of PGC-1α^22^. AMPK activation also suppresses inflammation^23^. Metrnl also increased AMPK activation by phosphorylation. Prior research revealed that irisin and PGC-1α/FNDC5 are expressed by the AMPK pathway. Additionally, by targeting AMPK, irisin reduces oxidative stress and inflammation in a variety of damage scenarios^24^.

It is has been proven that endurance exercise enhances the social communication and executive skills of children with ASD. Endurance exercises such as treadmill training, are known to use large muscle groups constantly and improve the aerobic capacity^17^.

These findings imply an interest in investigating the neuroprotective effects of treadmill training on ASD induced experimentally by valproic acid in mice, considering the role of MtD, oxidative stress, and inflammation in the pathophysiology of ASD. Our study was focused on investigating the role of myokines in modulating ASD-related manifestations.

## Materials and methods

### Animals

Forty male Swiss mice weighing 20–30 g were brought from Theodore Bilharz Research Institute (Giza, Egypt) and were acclimatized (5 mice/cage- two cages for each group). They were housed under conditions with regulated humidity, temperature, and 12-hour light/dark cycles. They were provided with unlimited access to water and ordinary mice chow. Animal care and use were approved by the Ethics Committee of the Faculty of Medicine-Menoufia University-Egypt with IRB NO: 8/2024 BIO12-1.

### Experimental design

The sample size was estimated using G*Power software (Aichach, Germany) as 40 mice. The study’s power was 80%, with a 95% confidence level. Every experiment was conducted in compliance with the US National Institutes of Health’s Guide for the Care and Use of Laboratory Animals (8th edition, National Academies Press) and the ARRIVE guidelines. After the acclimation phase, mice were randomly assigned to 4 groups with ten mice in each group.

**Fig. 1:**
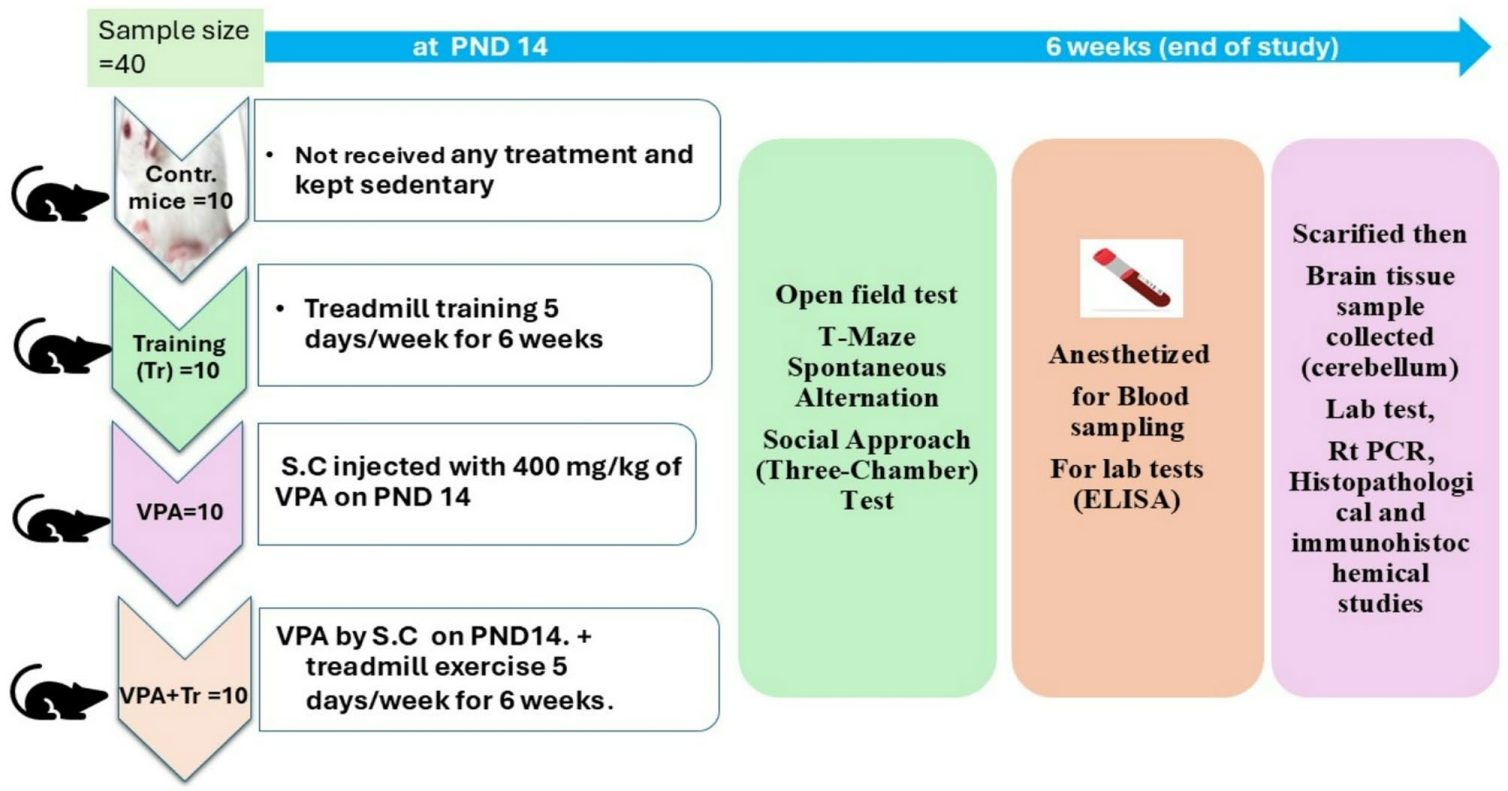
Schematic diagram of the experimental design: *Contr.,* Control group; *Tr,* Training group; *VPA,* Valproic acid group; *VPA + Tr* ,Valproic acid + Training; *S.C.,* subcutaneous; *PND 14*, Postnatal Day 14; *ELISA,* Enzyme immunoassay reaction; *RT-PCR,* real-time polymerase chain reaction.

1. **Control group**: Mice did not receive any treatment and were kept sedentary throughout the study period.
2. **Training group**: Mice were subjected to treadmill training 5 days/week for 6 weeks, as described below.
3. **Valproic acid-treated group (VPA)**: Mice were subcutaneously injected with 400 mg/kg of sodium valproic acid (Sigma Aldrich, St. Louis, MO, USA) once^25^ on the post-natal day (PND) 14 and left thereafter up to PND 56^26^.
4. **Valproic acid + Training group (VPA + Training)**: Mice received VPA on PND14. Then subjected to treadmill training 5 days/week for 6 weeks (Fig. 1).

### Exercise protocol

Because of its consistency, the treadmill training mechanism of endurance exercise was selected as the physical activity technique. All trained mice were permitted to adjust to treadmill running for 10 min on two consecutive days (first day at 5 m/min and second day at 8 m/min). Following the second familiarization phase, all the exercised mice will be subjected to incremental 30-min treadmill training protocol from (5 to 8 to 11 m/min) intensity at 0% slope for 6 weeks 5 day /week as in Table 1. After the exercise, mice returned to their cages with free access to food and water. Mice were not shocked while exercising on a treadmill^27^. The VO₂max was 35–45 mL/kg/min and this protocol of exercise is classified as moderate intensity^28^. Other mice spent the same amount of time on a treadmill that was switched off.

**Table 1:** Showing the training protocol in training and VPA + training groups.

Speed (m/min)	Time (min)
5	0–5
8	5–10
11	10–30

All mice were evaluated behaviorally 24 h after the intervention ended. They were then fasted for the entire night and had their retro-orbital blood samples taken for biochemical analysis. Finally, the euthanasia method was induced by xylazine and ketamine (10 and 35 mg/kg, respectively) anesthesia, then cervical dislocation and decapitation were done, followed by brain dissection. While the left half of the cerebellum was divided equally for biochemical analysis and reverse transcriptase polymerase chain reaction (RT-PCR) tests, 10% buffered formalin was used to preserve the right half for histopathological and immunohistochemical analysis.

### Neurobehavioral tests

Before testing, mice were given 30 min to get used to the behavior lab. The tests were conducted during the day, from 9 a.m. to 4 p.m., and at the same time every day. Using a timer, the researchers observed behavior in real-time. 70% ethanol was used to clean the test chambers for the subjects.

- **Open field test (OFT)**: The purpose of the exercise was to assess motor activity and stereotyped behaviors in an unfamiliar setting. The device was a 90 × 90 × 30 cm Plexiglas box with 16 identical squares divided into its floor. Each mouse was put in the center zone at the start of the test and allowed to freely explore the box. The video tracking system was used to record the number of crossing slots, rearing movements, and the times of center crossing for 5 min^29^.
- **T-maze spontaneous alternation test**: This test has been extensively used to examine both repetitive behavior and spatial working memory, which are key domains in autism. For ten consecutive trials, mice were placed on the T-maze base and permitted to move toward either the left or right arm. When all four paws were in one arm, it was considered an entrance^30^.
- **Social approach (Three-Chamber) test**: The cornerstone of this test is the innate tendency of animals to learn about a new environment. The three sessions are the habituation period, the sociability, and social novelty period. The 180 × 420 × 160 cm device is separated into three chambers that are joined by entryways. The left and right chambers each include a wire cage. Each mouse spent five minutes in the center of the middle chamber during the habituation period. Following that, Stranger 1, a mouse that had never interacted with the subject mouse before, was put in a wire cage in the right chamber. The subject mouse was then given ten minutes to investigate each chamber after the doorways were unlocked. The video monitoring device and a human observer who was blinded to the experiment groups recorded the time spent in each chamber (S1, S2) and the active interactions with stranger1 or the empty wire cage. During the third phase of this test, Stranger 2, a new unfamiliar mouse, was put in the other wire cage that was unoccupied. For ten minutes, the duration spent in each chamber and the time spent actively interacting with strangers 1 and 2 were recorded separately. The following formulas were used to determine the sociability index (SI) and social novelty index (SNI) during the second and third phases^29^:

$${\text{SI }}\left( {{\text{Sniffing}}} \right){\text{ }}=\frac{{{\text{Time exploring }}\left( {{\text{novel mouse 1}}} \right) - {\text{Time exploring }}\left( {{\text{novel object}}} \right)}}{{{\text{Time exploring }}\left( {{\text{novel mouse1}}} \right)\,+\,{\text{Time exploring }}\left( {{\text{novel object}}} \right)}}$$

$${\text{SNI }}\left( {{\text{Sniffing}}} \right)=\frac{{{\text{Time exploring }}\left( {{\text{novel mouse2 }}} \right)\, - \,{\text{Time exploring}}\left( {{\text{known mouse}}} \right)}}{{{\text{Time exploring }}\left( {{\text{novel mouse 2}}} \right)\,+\,{\text{Time exploring }}\left( {{\text{known mouse}}} \right)}}$$

$$\begin{gathered} {\text{SI (Chamber}}\;{\text{time) }} \hfill \\ {\text{=}}\frac{{{\text{Time spent in the chamber }}\left( {{\text{novel mouse1}}} \right) - {\text{Time spent in the chamber }}({\text{novel object}})}}{{{\text{Time spent in the chamber }}\left( {{\text{novel mouse1}}} \right)\,+\,{\text{Time spent in the chamber }}\left( {{\text{novel object}}} \right)}} \hfill \\ \end{gathered}$$

$$\begin{gathered} {\text{SNI (Chamber}}\;{\text{time)}} \hfill \\ {\text{= }}\frac{{{\text{Time spent in the chamber }}\left( {{\text{novel mouse 2}}} \right)\, - \,{\text{Time spent in the chamber }}\left( {{\text{known object}}} \right)}}{{{\text{Time spent in the chamber }}\left( {{\text{novel mouse 2}}} \right)\,+\,{\text{Time spent in the chamber}}\left( {{\text{known object}}} \right)}} \hfill \\ \end{gathered}$$

### Blood sample collection

Sera were separated after blood centrifugation at 3000 rpm for 15 min and stored at – 80 °C to be used for biochemical analysis of CRP, irisin, and metrnl levels using (CRP Mice ELISA kits (Abcam, ab22511, Cambridge, UK), (Mice irisin ELISA Kit Cat No. MBS7246352, MyBioSource, Sandiego, CA, USA), and (mice metrnl ELISA Cat. No. MBS8248450, MyBioSource, Sandiego, CA, USA) according to the manufacturer’s instructions.

### Tissue homogenate for biochemical analysis

To homogenize cerebellar tissue (10% w/v), ten milliliters of ice-cold Phosphate Buffer Saline (PBS) with a pH of 7.4 were utilized. For fifteen minutes, the homogenate was centrifuged at 4 °C and 4000 rpm.

The whole supernatant was utilized to measure cerebellar malondialdehyde (MDA) and catalase using specific colorimetric kits (Bio-diagnostic, Giza, Egypt) (MDA: Cat No. MD 25 29), (Catalase: Cat. No. CA 25 17). For the measurement of cerebellar TNF-α, interleukin (IL)-6, serotonin, citrate synthetase (CS) activity and BDNF, the following kits were utilized: (TNF-α: Cat No. ab208348, Abcam, Cambridge, UK), (IL-6: Cat No. ab222503; Abcam, Cambridge, UK), (Serotonin: Cat No. MBS160104, MyBiosource, Sandiego, CA, USA), (CS Activity Assay: Cat No. MBS8803743, MyBiosource, Sandiego, CA, USA), (Mice BDNF ELISA kits: Cat No. MBS355435, MyBiosource, Sandiego, CA, USA). Enzyme-linked immune sorbent assay kits (ELISA) were performed according to the manufacturer’s instructions.

### Quantitative assessment of AMPK, PPAR-ɣ, and Metrnl gene expressions using RT-PCR

Using the Qiagen RNeasy Plus Universal Mini-Kit (Cat No. 73404; Qiagen, USA), total RNA was extracted from cerebellar tissues. With a Nanophotometer N60, the RNA’s quality and purity were evaluated. Until further use again, the extracted RNA was kept at -80 °C. Using an Applied Biosystems 2720 Singapore heat cycler and the QuantiTect Reverse Transcription Kit (Cat No. 205311; Qiagen, USA), cDNA was synthesized in the first stage. The DNA Wipeout Buffer, Quantiscript Reverse Transcriptase, Quantiscript RT Buffer, RT Primer Mix, and RNase-free water were thawed at room temperature (15–25 °C) and then stored on ice, while the template RNA was thawed on ice. With a total amount of 14 µl (5 µl RNA template, 2 µl gDNA Wipeout Buffer, and 7 µl nuclease-free water), the genomic DNA elimination reaction was generated in a sterile, nuclease-free micro-Eppendorf on ice, mixed, and preserved on ice. After two minutes of incubation at 42 °C, the mixture was promptly cooled on ice. One microliter of Quantiscript Reverse Transcriptase, four microliters of Quantiscript RT Buffer (5×), and one microliter of RT Primer Mix were added to create the reverse-transcription master mix, which had a total volume of 20 µl. After 15 min of incubation at 65 °C, the micro-Eppendorf was incubated for 3 min at 95 °C to stop the reaction. Until additional analysis, the resultant RT reaction products were kept at -20 °C.The QuantiTect SYBR Green PCR Kit (Cat No. 204143; Qiagen, USA) was utilized in SYBR Green-based quantitative real-time PCR for the subsequent cDNA amplification step. 10 µl of SYBR Green PCR Master Mix, 3 µl of cDNA, 1 µl of each forward and reverse primer, and 5 µl of RNase-free water were used to construct the PCR reaction, which had a total volume of 20 µl. A 15-minute activation stage at 95 °C was used to start the process, which was then followed by 45 cycles. Each cycle had three stages: a 30-second annealing phase at 60 °C, a 30-second extension phase at 72 °C, and a 15-second denaturation phase at 94 °C **(**Table 2**).**

**Table 2:** Primers used for measuring the expression of AMPK, PPAR-ɣ, Metrnl and GAPDH genes.

AMPK	Forward: 5’- TGCTACTCCACAGAGATCGG−3’ Reverse: 5’- GTCTGAGGGCTTTCCTTGAG-3’
PPAR-ɣ	Forward: 5’- TCAGCTCTGTGGACCTCTCC-3’ Reverse: 5’- ACCCCTTGCATCCTTCACAAG-3’
Metrnl	Forward: 5’-ACCAGTGACTTTGTTGTCCGA-3’ Reverse: 5’-CACCCGCAGGTAGATGACTG-3’
GAPDH	Forward: 5’- GGAGAGTGTTTCCTCGTCCC-3’ Reverse: 5’- ATGAAGGGGTCGTTGATGGC-3’

The comparative ∆∆Ct method was used to evaluate the RQ of the AMPK, PPAR-ɣ, and mertnl genes^31^, in which the target genes’ mRNA levels were compared to a calibrator after being adjusted to an endogenous reference gene (GAPDH). The specificity and identity of the PCR products were confirmed by melting curve analysis. The melting curve program comprised 15 s at 95 °C, 1 min of fluorescence data gathering at 60 °C, and 1 s at 95 °C. Version 2.0.1 of the Applied Biosystem 7500 software was used to analyze the data.

### Histopathological, immunohistochemical, and quantitative assessment

Following a 24-hour fixation in 10% neutral buffered formalin, the cerebellum was dehydrated using increasing alcohol grades, cleaned, and embedded in paraffin blocks. Serial tissue slices were deparaffinized, cut to a thickness of 5 μm, and stained with eosin and hematoxylin^32^.

**Immunohistochemical examination**: After being deparaffinized, the 5-µm brain paraffin slices were rehydrated using decreasing alcohol grades. Sections were placed in 3% hydrogen peroxide (H_2_O_2_) to inhibit endogenous peroxidase. A protein blocker was used to block nonspecific binding sites, and then the primary antibody anti-GFAP (Rabbit polyclonal, ab7260, 1:300, Midco Trade, Giza, Egypt), anti-Bax (rabbit polyclonal, Sigma-Aldrich, Cairo, Egypt), and a rabbit polyclonal anti-PPAR-γ (1:300 dilution; Santa Cruz Biotechnology, Santa Cruz, CA) were added with overnight incubation. After that, a biotinylated goat-polyvalent secondary antibody at a concentration of 2% (Vector, Peterborough, UK) was applied for 10 min (37 °C), and then the avidin-biotin-peroxidase complex (Vector) was added^33^.

**For quantitative assessment**, Using Image J software (1.74v), five distinct section (400 X) from a minimum of five mice per group were examined to determine the number of PCs and the area percentage of GFAP, Bax, and PPAR-γ.

### Data management

Statistical analysis was performed using Statistical Package for Social Sciences (SPSS) version 28 (SPSS Inc., Chicago, IL, USA). Both descriptive and inferential statistical methods were used in the data analysis process. Number (No.), percentage, mean (x̅), and standard deviation (SD) were used to represent descriptive statistics. Analytical statistics included paired samples, the ANOVA with repeated measures test to compare the four-group means of the three-chamber test, where the mice in each group are identical, and the one-way ANOVA test (parametric) followed by a post hoc test for quantitative data. The t-test was used to compare two group means for the same group at different intervals. Statistical significance was defined as a P value < 0.05.

## Results

### Open field test (OFT)

**Fig. 2:**
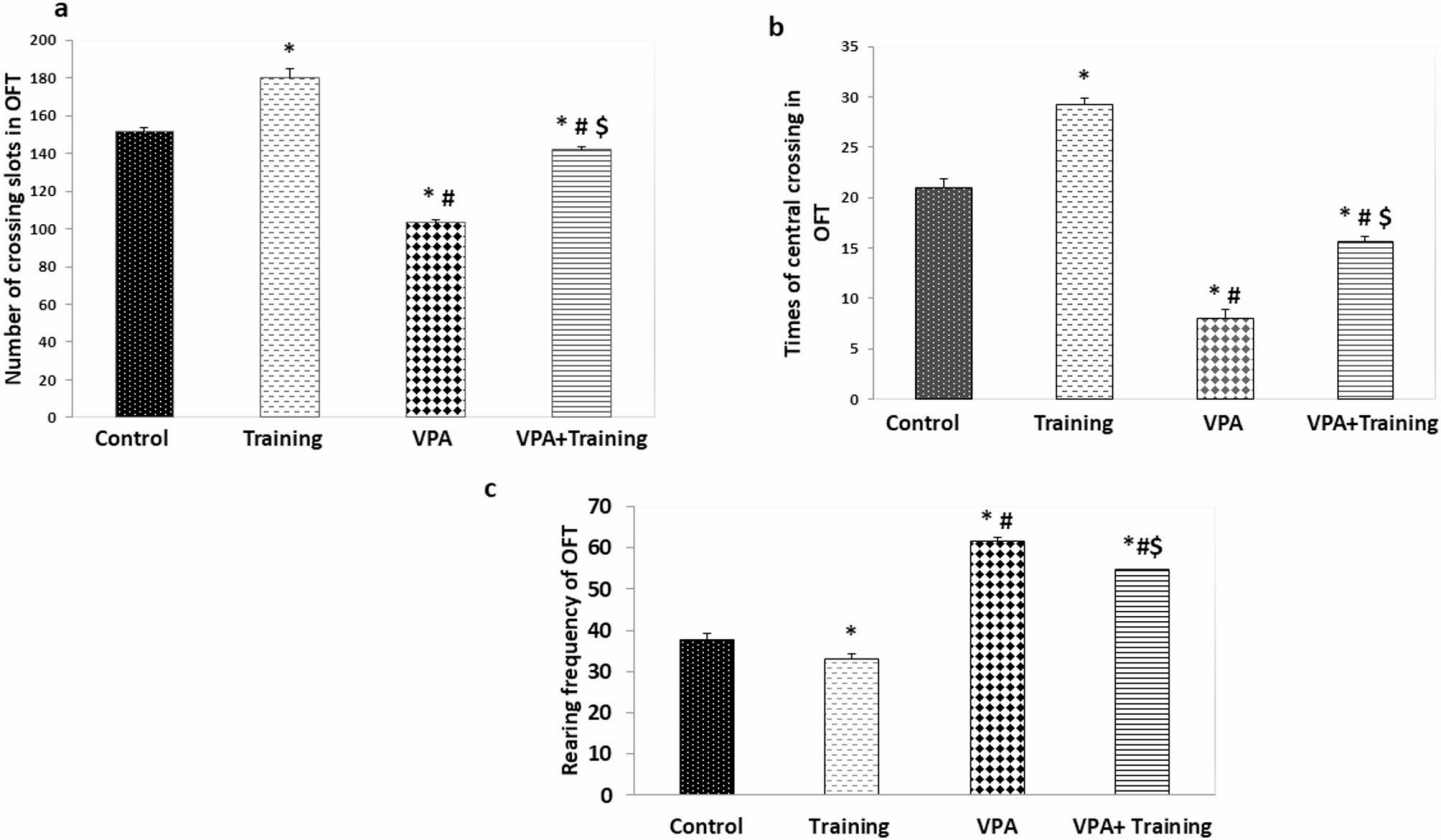
The number of crossing slots, times of central crossing, and rearing frequency in open field test in the studied groups. *Significant compared to the control group. ^#^Significant compared to the Training group. $ Significant compared to VPA-group.

The number of crossed slots was found to be markedly decreased in the VPA group in comparison to the control and Training groups (103.6 ± 1.9 vs. 151.8 ± 23.5, 180.3 ± 4.3, respectively; *P* = 0.000). Conversely, it was substantially increased in the VPA + Training group than that of the VPA group (141.8 ± 1.47; *P* = 0.000). Also, the times of central crossing by mice was found to be dramatically decreased in the VPA group compared to the controls (7.9 ± 0.87 vs. 21.0 ± 0.87), and the training group (29.2 ± 0.56) (*P* = 0.000). Conversely, it was substantially increased in the VPA + Training group than that of the VPA group (15.7 ± 0.48; *P* = 0.000). However, it remained considerably diminished compared to control group (*P* < 0.05), and Training group (*P* = 0.00). Rearing frequency was found to be substantially elevated in the VPA compared to the control and Training groups (61.6 ± 0.96 vs. 37.7 ± 1.4, and 33.2 ± 0.63, respectively; *P* = 0.000). Conversely, it was considerably decreased in the VPA + Training group than that of the VPA (54.6 ± 1.03; *P* = 0.000). However, it remained markedly increased compared to the control and Training groups (*P* = 0.000) (Fig. 2).

### Social approach (three-chamber) test

Session 1 showed that the time spent by the subject mouse in the empty chamber was considerably less than that of the control group’s subject mouse with stranger (100.11 ± 1.63 vs. 454.83 ± 3.31 sec, respectively) and Training group (101.5 ± 1.23 vs. 494.2 ± 1.56 sec, respectively) (*P* = 0.000).

However, mice in the VPA group spent almost the same time in the stranger and empty rooms, indicating that they did not prefer social closeness (101.9 ± 1.87 vs. 107.2 ± 1.41 sec, respectively). In the VPA + Training group, there was a substantial increase (*P* = 0.000) in the time spent with the stranger mouse concerning the time spent in the empty chamber (289.83 ± 3.31 vs. 116.5 ± 2.36 s, respectively).

**Fig. 3:**
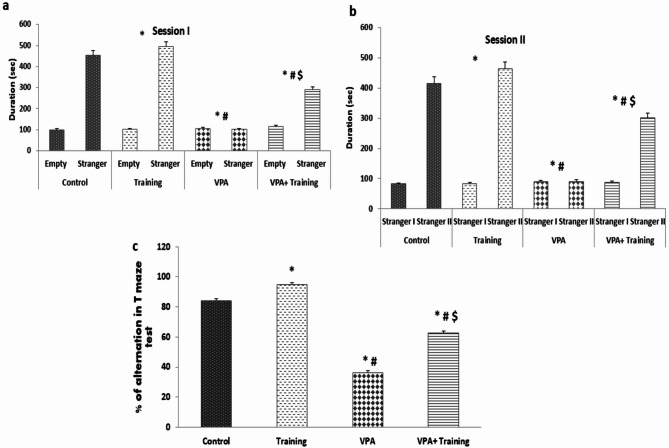
Social approach (three-chamber) test and % of alteration in T-maze alteration test in the different studied groups. *Significant compared to the control group, ^#^Significant compared to the Training group. $ Significant compared to VPA-group.

In session two, the subject mice in the control group spent considerably more time with stranger II than with stranger I (415.3 ± 3.6 vs. 82.4 ± 2.06 sec). The Training mice in the training group spent significantly more time with stranger II than with stranger I (463.7 ± 3.6 vs. 83.56 ± 2.06 sec). However, mice in the VPA group spent almost the same amount of time in the strangers I and II chambers (91.02 ± 1.41 vs. 89.9 ± 1.47 sec, respectively), indicating that they did not prefer social proximity. The amount of time spent with stranger II in the VPA + Training group was much longer than that spent in the stranger I chamber (301.11 ± 2.78 vs. 88.30 ± 1.87 sec, respectively) but was significantly decreased compared to Training mice (Fig. 3).

### T-maze alteration test

The VPA group’s percentage of alteration was lower than that of the control and Training groups in the T-maze alteration test (36.2 ± 1.31 vs. 78.0 ± 1.73, 84.0 ± 1.33%, respectively; *P* = 0.000). However, it was significantly increased in the VPA + Training group than in the VPA mice (62.5 ± 1.43%; *P* = 0.000). Nonetheless, it remained considerably lower than the control and the Training mice (*P* = 0.000) (Fig. 3).

**Fig. 4:**
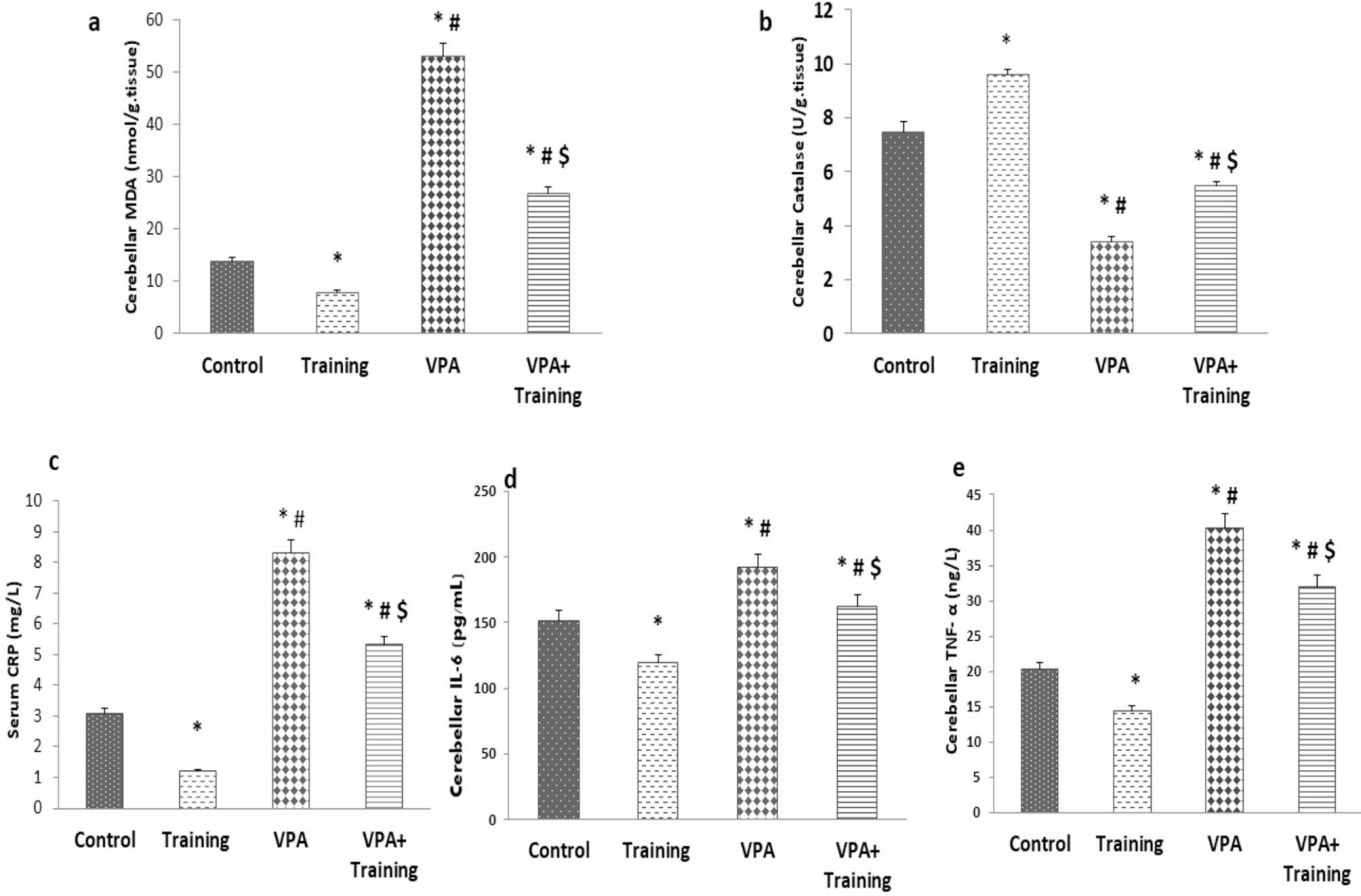
The oxidative stress markers (cerebellar MDA and Catalase), and the inflammatory markers (serum CRP, cerebellar IL-6, and TNF- α) in the studied groups. *Significant in comparison to the control group. ^#^Significant compared to the Training group. $ Significant compared to VPA-group.

Regarding biochemical assessments, in the VPA group, the cerebellar MDA level was dramatically elevated than in the control and Training groups (53.06 ± 1.12 vs. 13.83 ± 0.88, 7.8 ± 0.24 nmol/g. tissue, respectively, (*P* = 0.000). In the VPA + Training group, it was considerably diminished compared to the VPA group (26.80 ± 0.39 nmol/g. tissue *P* = 0.000). However, it remained substantially elevated than the control, and Training groups (*P* = 0.000). In comparison to the control and Training groups, the VPA group’s cerebellar catalase level was considerably lower (3.43 ± 0.16 vs. 7.49 ± 0.35, 9.6 ± 0.16 U/g. tissue, respectively; *P* = 0.000). In contrast, the VPA + Training group’s value was higher than the VPA group’s (5.50 ± 0.14 U/g. tissue *P* = 0.000). However, it was still markedly lower than control and Training groups (*P* = 0.000) (Fig. 4).

The VPA group’s serum CRP level was considerably higher compared to control and Training groups (8.3 ± 0.23 vs. 3.09 ± 0.12, 1.22 ± 0.31 mg/L, respectively; *P* = 0.000). It was substantially decreased in the VPA + Training group (5.3 ± 0.18 mg/L; *P* = 0.000). It was still much higher than the control and training groups. The VPA group had a considerably higher level of cerebellar IL-6 than the control and Training groups (192.5 ± 1.09 vs. 151.5 ± 1.04vs.119.6 ± 1.21pg/ml, respectively; *P* = 0.000). In contrast, it was dramatically lowered in the VPA + Training group (162.6 ± 1.08 pg/ml; *P* = 0.000) than in the VPA group. However, it was still higher than the control and Training groups (*P* = 0.000). Additionally, the VPA group’s cerebellar TNF-α level was considerably higher than the control mice (40.28 ± 1.05 vs. 20.21 ± 0.89, 14.38 ± 0.26 ng/L, respectively; *P* = 0.000). It was considerably lower in the VPA + Training group than in the VPA group (32.03 ± 0.89 ng/L; *P* = 0.000). However, it remained considerably higher than the control and Training groups (*P* = 0.000) (Fig. 4).

**Fig. 5:**
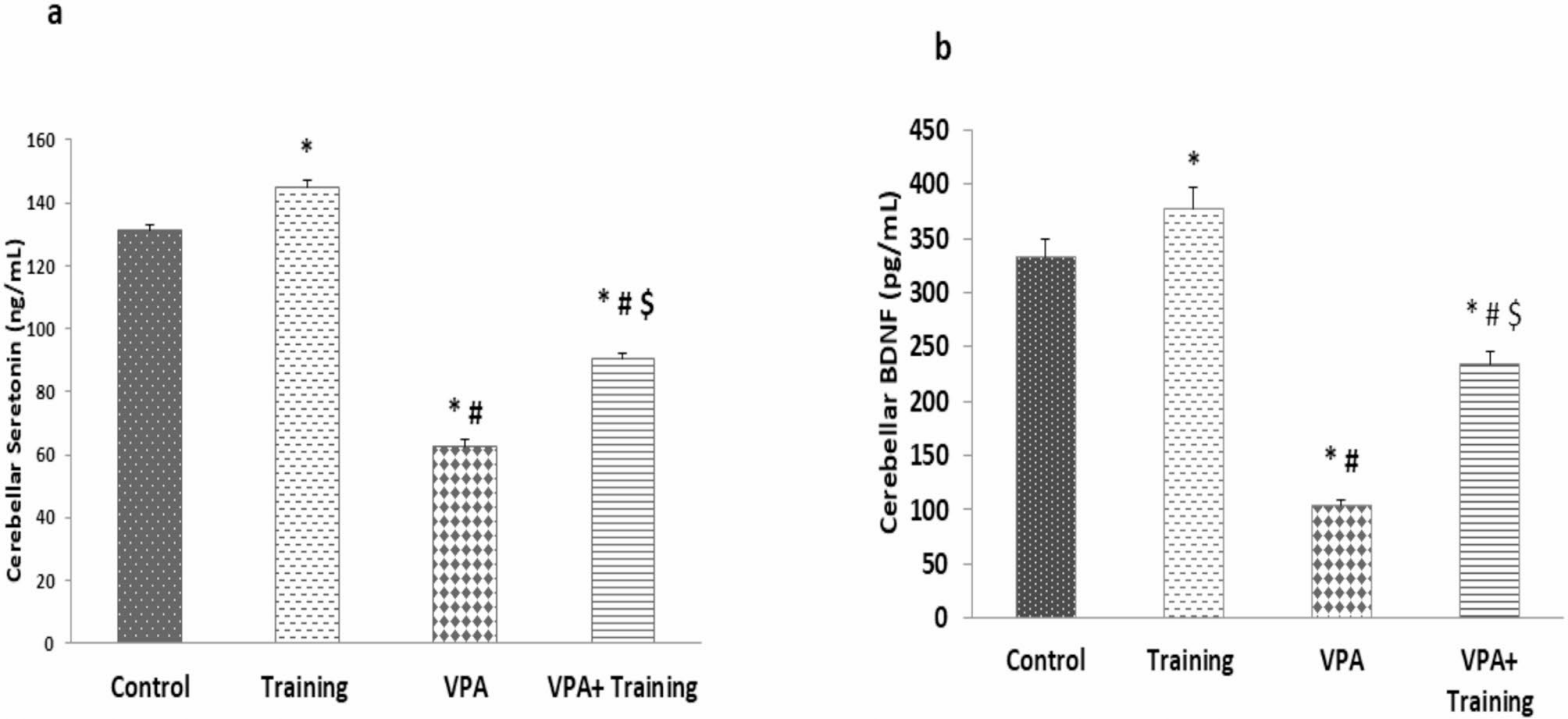
Showing the effect of training on cerebellar serotonin and BDNF in the studied groups. *Significant compared to the control group, ^#^Significant compared to the Training group. $ Significant compared to VPA-group.

In terms of neurotransmitter evaluation, the VPA group’s cerebellar serotonin level was considerably lower than those of the control and Training groups (62.83 ± 2.31 vs. 131.17 ± 1.72, 145.1 ± 2.1, ng/ml, respectively; *P* = 0.000). However, compared to the VPA group, it was markedly higher in the VPA + Training group (90.20 ± 1.76 ng/ml; *P* = 0.000). However, it was still lower than the control, and training groups (*P* = 0.000). Cerebellar BDNF was shown to be considerably lower in the VPA group than in the control and Training groups (104.4 ± 5.91 vs. 333 ± 7.31, 378 ± 2.69 pg/ml, respectively; *P* = 0.000). However, compared to the VPA group, it was statistically higher in the VPA + Training group (233.7 ± 33.67 pg/ml; *P* = 0.000). However, it was still lower than the control and Training mice (*P* = 0.000) (Fig. 5).

**Fig. 6:**
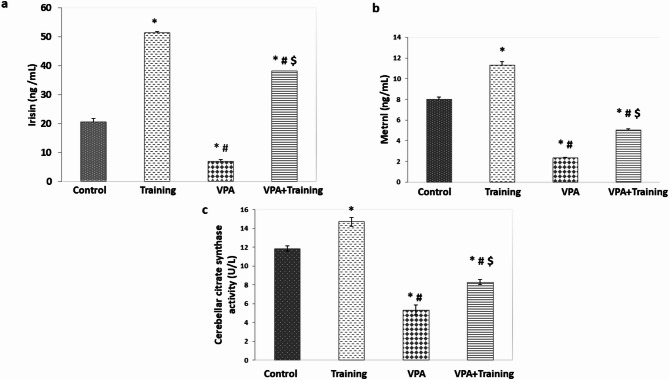
Training impact on the studied groups’ levels of serum irisin, metrnl, and cerebellar citrate synthetase activity.*significant in comparison to the control group. ^#^Significant compared to the Training group. $ Significant compared to VPA-group.

Compared to the control and Training group, the VPA group’s serum irisin level was considerably lower (6.8 ± 1.2 vs. 20.79 ± 1.01, 51.5 ± 1.26 ng/mL, respectively; *P* = 0.000). In contrast, the VPA + Training group’s level was statistically higher than the VPA group’s (38.26 ± 0.68 ng/mL; *P* = 0.000). However, it was still lower than the Training group and higher than the control group (*P* = 0.000) (Fig. 6).

Serum levels of metrnl were considerably lower in the VPA group than in the control and training groups (2.33 ± 0.08 vs. 8.0 ± 0.23, 11.3 ± 0.73 ng/mL, respectively; *P* = 0.000). It was considerably higher in the VPA + Training group than in the VPA group (5.01 ± 0.11 ng/mL; *P* = 0.000). However, it still lower than control and Training groups (*P* = 0.000) (Fig. 6).

Additionally, the VPA group’s citrate synthase level was lower than that of the control and training group (5.31 ± 0.27 vs. 11.83 ± 0.45, 14.7 ± 0.52 U/L, respectively; *P* = 0.000). But compared to the VPA group, it was statistically higher in the VPA + Training group (8.28 ± 0.29 U/L; *P* = 0.000). However, it was still lower than the training and the control groups (*P* = 0.000) (Fig. 6).

**Fig. 7:**
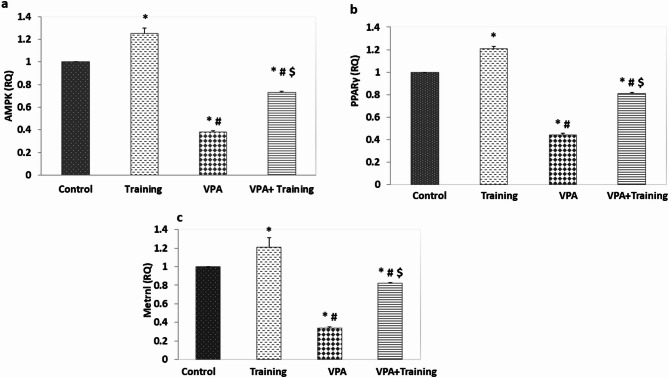
The effect of training on the expression of the cerebellar AMPK/ PPAR-γ/ metrnl genes in the studied groups. *Significant compared to control, ^#^Significant compared to the Training group. $ significant compared to VPA-group.

The VPA group’s AMPK gene expression level was downregulated than that of the control and Training groups (0.37 ± 0.01 vs. 1.0 ± 000, 1.25 ± 0.02, respectively, *P* = 0.000). It was considerably more prevalent in the VPA + Training group than in the VPA group (0.72 ± 0.01; *P* = 0.000). When compared to the control and Training groups, the AMPK level was still considerably down regulated (*P* = 0.000). Additionally, the VPA group’s PPAṚ-γ level was considerably lower than that of the control and Training groups (0.44 ± 0.01 vs. 1.0 ± 0.001, 1.28 ± 0.04, respectively, *P* = 0.000). PPAR-γ gene expression was considerably more prevalent in the VPA + Training group than in the VPA group (0.81 ± 0.01; *P* = 0.000). However, it remained considerably down regulated compared to the control and Training groups, (*P* = 0.000) (Fig. 7).

The level of metrnl gene expression was considerably down regulated compared to the control and Training groups (0.34 ± 0.01 vs. 1.0 ± 0.00, 1.2 ± 0.03 respectively; *P* = 0.000). Metrnl was considerably up regulated in the VPA + Training group than VPA group (0.81 ± 0.01; *P* = 0.000). In contrast to the control, and Training groups, metrnl gene expression was nevertheless considerably down regulated (*P* = 0.000) (Fig. 7).

### Histopathological results

#### H&E staining of the cerebellum

The three layers that make up the control group’s cerebellum are the inner granular layer (GL), middle Purkinje cell layer (PC), and outer molecular layer (ML). The neuropil with stellate and basket cells is intact in the ML. One row of flask-shaped Purkinje cells (PCs) with apical dendrites, large nucleoli, and rounded vesicular nuclei is visible in the PCL. Numerous distinct, rounded cells with highly stained, rounded nuclei and sparse, acidophilic cytoplasm, interspersed with cerebellar islands, are visible in the GL. The VPA group displayed ML vacuolation. PCL displays a disrupted linear PC layout. Some PCs looked deteriorated and smaller, while others were completely lost, leaving empty spaces. Pyknotic nuclei can be seen in the GL. Apart from a few deteriorated PCs among the normal ones, the VPA + Training group appeared almost normal.

**Fig. 8:**
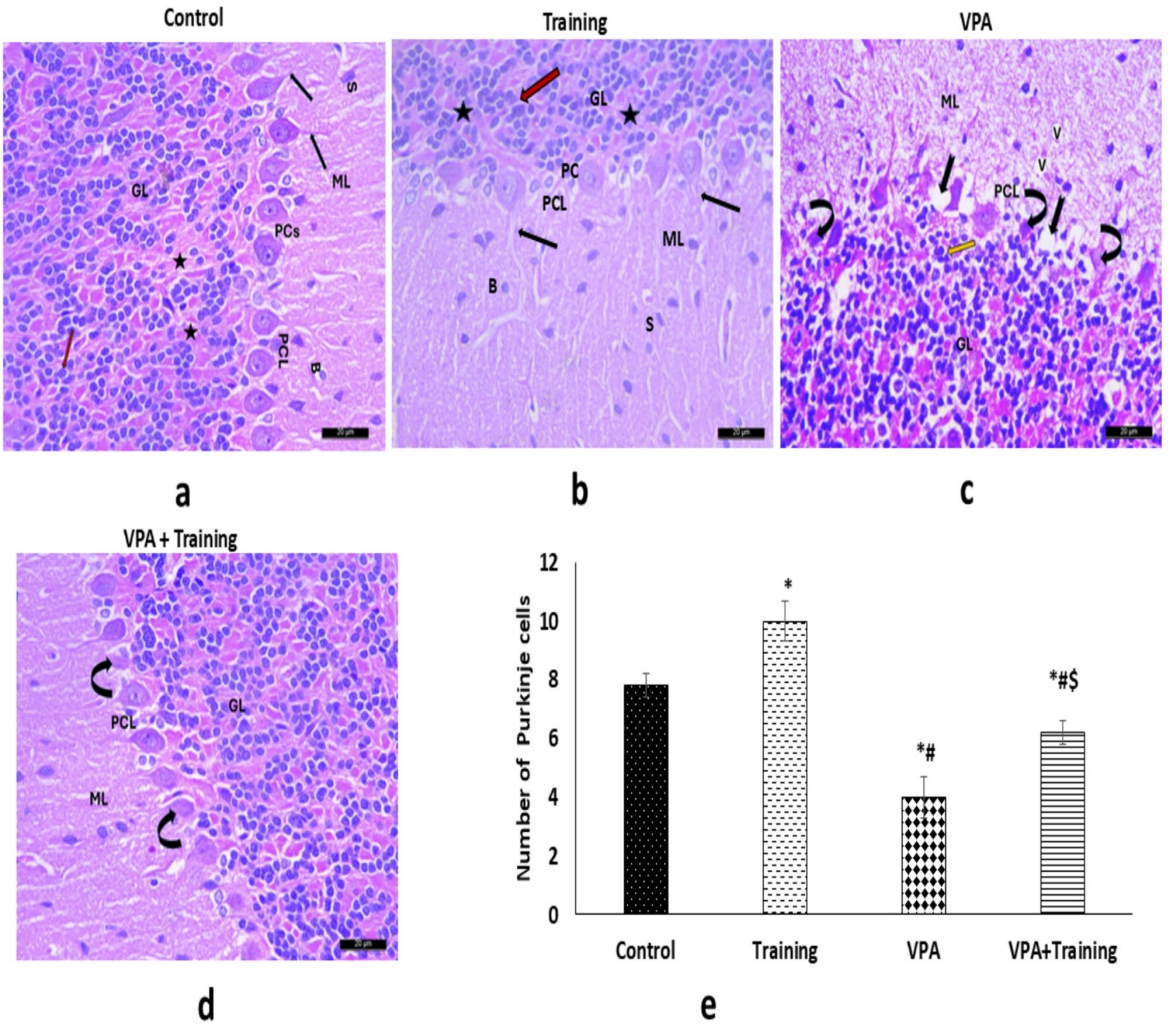
H&E-stained cerebellar slices from each group are shown in representative photomicrographs (×400, scale bar = 20 μm) along with a Purkinje cell loss count. The three layers of the normal cerebellum- the inner granular layer (GL), middle Purkinje cell layer (PCL), and outer molecular layer (ML)- make up the control, and Training groups (**a**, **b**). The ML showed intact neuropil with stellate (S) and basket cells (B). One row of flask-shaped Purkinje cells (PCs) with apical dendrites (black arrows), large nucleoli, and rounded vesicular nuclei was visible in the PCL. GL revealed rounded cells with cerebellar islands (stars) in between and rounded, darkly stained nuclei (red arrow). (**c**): VPA group: vacuolation of the ML (V), disturbed linear arrangement of PCs, some cells appeared degenerated (arched arrows), others are completely lost leaving empty spaces (notched arrows). The GL shows the pyknotic nuclei of its cells (yellow arrow). (**d**): VPA + Training group: degeneration of few PCs. (**e**) Number of PCs. ∗Significant versus the control group. ^#^Significant versus the Training group. $ Significant versus VPA-mice.

In the VPA group, there were substantially fewer PCs than in the control and Training groups (4.8 ± 0.83 vs. 8.4 ± 0.54, 10.32 ± 0.25, *P* = 0.00). While the number of PCs was much lower than the control group, it was upregulated in the VPA + Training group compared to the VPA group (6.4 ± 0.54 vs. VPA, *P* = 0.000) (Fig. 8).

#### **Immunohistochemical staining of the cerebellum**

**Fig. 9:**
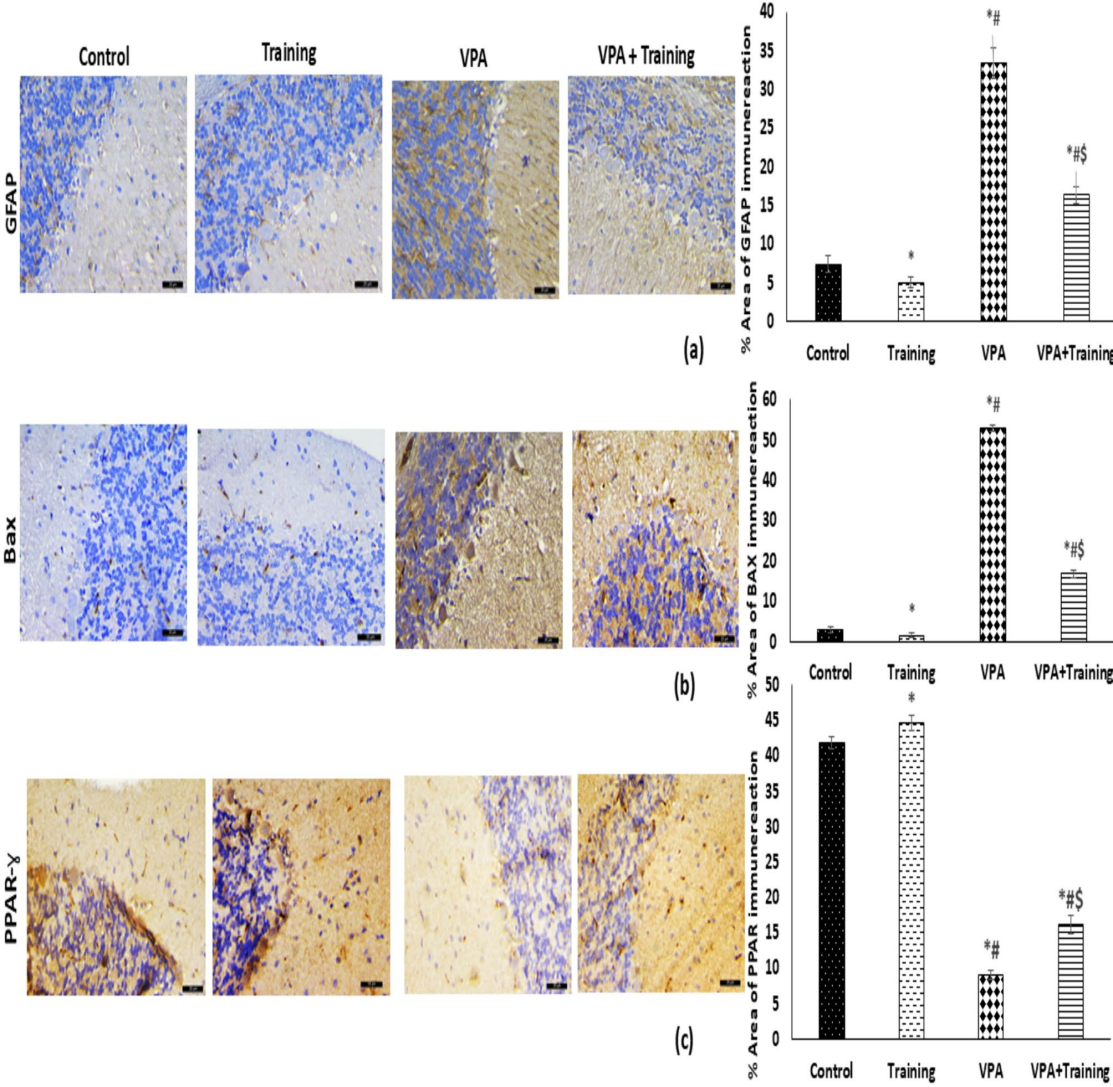
Quantitative analysis of positive immunoreaction and representative photomicrographs of the cerebellar immunostaining of the various groups (×400, scale bar = 20 μm). GFAP (a), Bax (b), and PPAR-γ (c). In comparison to the control and Training groups, the VPA group exhibits downregulation of PPAR-γ immunoreaction but overexpression of GFAP and Bax. In comparison to VPA, the VPA + Training group exhibits overexpression of PPAR-γ but downregulation of GFAP and Bax. However, when compared to the control and Training group, it was much lower in PPAR-γ but significantly higher in GFAP and Bax. ∗Significant versus control group ^#^Significant versus the Training mice. ^$^Significant versus VPA-mice.

**Immunohistochemical Assessment of GFAP**,** Bax**,** and PPAR-**γ **Immunoreaction**:

VPA treated group showed a dramatic increase in the percentage of the GFAP compared to the control and Training groups (33.2 ± 2.3vs 7.4 ± 1.1, and 5.34 ± 0.02, respectively; *P* = 0.000), and also marked increase in Bax compared to the control and Training groups (53.2 ± 1.9 vs. 3 ± 0.85, and 2.45 ± 0.07, respectively; *P* < 0.050), but there was a dramatic decrease in PPAR-γ immunoreaction compared to the control, and Training groups (9 ± 0.7 vs. 41.8 ± 1.7, and 43.9 ± 1.04, respectively; *P* = 0.000). In contrast, the VPA + Training group exhibited a significant increase in PPAR-γ immunoreaction (16.2+1.3;P=000), but a significant decrease in GFAP (16.4 ± 1.1; *P* = 0.000) and Bax (16.8 ± 0.8; *P* = 0.000) when compared to the VPA treated group. However, when compared to the control and Training groups, it was much lower in PPAR-γ but significantly higher in GFAP and Bax (Fig. 9).

## Discussion

Despite slight improvements in the evidence supporting ASD therapy, the available remedies are not enough to reduce the symptoms of ASD. Our results showed that mice that received VPA on the PND 14 in a dose of 400 mg/kg exhibited autism-like symptoms, including social impairment, anxiety, reduced exploratory activity, and impaired brain cognitive function. VPA group also showed significant disturbance of the oxidative balance and brain enzyme activity accompanied by an inflammatory state. Down-regulation of metrnl, AMPK, and PPAR-γ genes in the cerebellum were also observed to be involved in ASD pathophysiology. Treadmill training ameliorated all VPA-induced changes in the cerebellar tissue of autistic mice denoting its positive impact on VPA-induced ASD.

Training group dramatically demonstrated significant elevation in the antioxidant parameters, enhanced mitochondrial activity, reduced inflammatory cytokines and elevated cerebellar serotonin level and serum irisin level denoting positive impact of training in ameliorating oxidative stress, inflammation and enhancing mitochondrial activity.

Postnatal valproic acid is a useful model for investigating the neurochemical and behavioral changes in autism to find new therapies. The hippocampus, striatum, and cerebellum undergo neuronal migration, differentiation, myelination, synaptogenesis, and glio-genesis during the crucial time of PND 14^6^.

VPA significantly reduced crossing slots and center entrance times in the open field test and significantly increased rearing movements when compared to the control mice. These results went hand by hand with other studies that discovered that giving VPA to the PND 14 resulted in autism-like symptoms, including anxiety, reduced exploratory activity, and impaired brain cognitive function. VPA might change the amounts of gene transcription, which might lead to synaptic dysfunction and impairments in neurogenesis^34^.

Social interaction is one of the quantitative markers used to diagnose ASD^35^. Utilization of the three-chambers test and the T-maze test, the VPA group’s results demonstrated significant impairment in social interaction behavior and a reduction in spontaneous alternation tasks, respectively. These results were agreed with earlier published studies that showed that exposure to VPA was linked to a decrease in socializing language and repetitive, stereotyped behaviors^36^. VPA may decrease neuronal activity, raise the inhibitory neurotransmitter GABA in the brain, and change the neural circuits that lead to ASD by blocking sodium-calcium channels^37^. Furthermore, VPA functions by inhibiting the activity of histone deacetylase, which modifies DNA structure and impacts synaptic plasticity. In several parts of the brain, particularly the cerebellum, this results in apoptotic cell death, which affects behavior and memory^38^.

VPA implementation-induced changes in memory, social and exploratory behavior, and neurobehavioral processes were all significantly enhanced by using treadmill training. Despite our findings, prior research has demonstrated that exercise might help with social behavior issues, anxiety, and memory impairments, making it a potentially promising treatment for ASD^39^.

Since early identification and treatment of ASD have been demonstrated to greatly improve ASD outcomes and minimize impairment, finding precise biomarkers can be very useful in clinical practice. The biochemical profile of the VPA group showed a considerable rise in brain MDA level, a lipid peroxidation marker, and a significant decrease in brain antioxidant catalase concentration when compared to the control group. These findings align with those of prior research^40^. Growing body evidence connected brain oxidative stress with MtD in ASD. Chauhan et al. reported that VPA increased cerebellar MDA, which was the result of a chain reaction combining polyunsaturated fatty acids and dangerous ROS^41^. Antioxidant proteins were essential ROS scavengers. Therefore, raised ROS production and/or ineffective antioxidant metabolism are linked to ASD^42^. Furthermore, oxidative stress led to inflammatory changes, the breakdown of lipids, proteins, and DNA, as well as damage to brain tissue that may culminate in ASD^43^.

Treadmill training reduces oxidative stress by stimulating physiological and biochemical processes in all body organs, especially by raising skeletal muscle energy expenditure. Since it impacts mitochondria, which are the primary site of ROS, and since it activates the Nrf2 gene, which raises the transcription of antioxidant genes, treadmill exercise may have antioxidant benefits^44^**.** Besides, training increases brain metabolism, neurogenesis, and neuronal activity, according to Quan et al. This was linked to changes in the redox-sensitive signaling pathway and improved antioxidants^45^. Our results support the previous studies, as treadmill training restored the oxidative balance by inducing a noticeable improvement in MDA and a noticeable increase in catalase levels in the VPA + Training group compared to the VPA group.

Akintunde et al. stated that VPA was a pro-inflammatory substance that could lead to neuroinflammation and/or mitochondrial dysfunction, both of which can cause ASD^46^. The current study validates the previous research since VPA significantly increased inflammatory markers (CRP, IL-6, and TNF-α) in the VPA group versus the control mice. Higher plasma levels of the pro-inflammatory cytokines TNF-α and IL-6 were linked to the severity of ASD^47^. IL-6 has a major impact on the genesis of ASD and might provide a molecular marker to help with early detection. Both ASD and cerebellar demyelination were caused by elevated IL-6. Furthermore, both excitatory and inhibitory synaptic forms are altered by the increase in IL-6 caused by VPA, leading to aberrant changes in dendritic spine length, shape, and distribution pattern as well as synaptic transmission^48^. It has been found that neuroinflammation in the prefrontal cortex causes patients with ASD to exhibit poorer social behavior and less exploratory activity^49^.

The current study showed that treadmill training significantly reduces neuroinflammation caused by VPA therapy. Reduced visceral fat mass and increased skeletal muscle synthesis and release of anti-inflammatory cytokines, which in turn limited the generation of pro-inflammatory cytokines, have been the main mechanisms by which exercise reduces inflammation^50^.

Our histological results showed Purkinje cell (PC) degradation and a significant cell loss in the VPA group. Galal et al. had connected VPA to increased levels of free radicals and degenerative changes in the cerebellum^51^. Conversely, trained mice in this study showed a significant increase in the quantity of PCs. The antioxidant and anti-inflammatory effects of exercise may contribute to this discovery. Training on a treadmill improved PC’s survival rate in the cerebellar vermis following traumatic brain injury^52^.

In the present study, the cerebellar cortex of the VPA-treated group showed noticeably higher levels of GFAP expression. However, treadmill activity reduced the expression of GFAP in the autistic mice. Conversely, Purkinje neurons were more likely to survive when the autistic mice engaged in treadmill activity because it inhibited reactive astrocytes and microglia. This was consistent with Lee et al.'s findings^53^. Additionally, GFAP expression and astrocytic dysfunction reduced neuronal survival and were connected to detrimental circumstances in the central nervous system^54^. Ramesh et al. claimed that astrocytes release cytokines after reactive activation, which exacerbates the inflammatory response and causes more brain damage^55^.

Cerebellar apoptosis was revealed by the up-regulated Bax pro-apoptotic protein in the VPA group. This is explained by the neuro-inflammation and oxidative stress that VPA causes, which leads to cell death. VPA increased Bax and decreased Bcl-2 (anti-apoptotic) through the activation of the intrinsic mitochondrial apoptotic pathway^56^. Biochemical investigations demonstrated alterations in the levels of p53 and Bax/Bcl-2 in the parietal and cerebellar areas of autistic brains^57^**.**

Conversely, the trained group showed down regulated Bax, indicating that exercise had antiapoptotic properties. Training significantly decreased PC apoptosis by improving nerve myelination, reducing inflammatory conditions, and restoring oxidative balance^58^.

In our investigation, we found that the cerebellum of the VPA group had significantly lower levels of serotonin. This finding was consistent with several researchers’ findings that VPA may affect the serotonergic system directly or indirectly^59^**.** Because high blood levels of serotonin during early brain development caused the brain to lose serotonin terminals in a negative feedback system, people with ASD had lower serotonin concentrations. Early brain development uses serotonin as a growth agent, which has been shown to have a detrimental effect on social behavior^60^**.** Disturbances in neurotransmitter systems, such as those involving serotonin and dopamine, are among the main causes of stereotyped behaviors in ASD^61^. On the other hand, Treadmill training was able to up-regulate the cerebellar serotonin expression. This result was consistent with other studies^62^**.**

One of the primary causes of oxidative stress is mitochondria. Enzymes that are strictly controlled typically produce ROS. By generating an excessive amount of ROS, overstimulation of the electron transport chain and NAD(P)H results in oxidative stress^63^. The pathophysiology of ASD was influenced by MtD as the brain is extremely sensitive to any decrease in mitochondrial efficiency because of its high aerobic metabolic demand^8^.

The enzyme mitochondrial citrate synthase is the pacemaker in the Krebs cycle and a distinct marker of the mitochondrial matrix. The quantity and activity of citrate synthase indicate the presence of intact mitochondria^9^. The current investigation found that the cerebellar citrate synthase enzyme activity in the VPA group was considerably lower than that of the control group. VPA inhibits the citrate synthetase enzyme and respiratory electron transport chain complexes^64^. Citrate synthetase enzyme reduction impacts the mitochondrial membrane’s conversion of acetyl CoA to citrate, which diminishes Ach, a chemical role in memory and cognition^65^. Training, on the other hand, markedly raised mitochondrial citrate synthetase. This outcome was in line with the findings of previous research^66^.

Movement and posture were formerly believed to be the primary determinants of skeletal muscle function. However, new research has revealed that myofibers react to exercise by expressing and releasing a range of paracrine and endocrine-acting chemicals, including irisin, and metrnl. During training, skeletal muscle and adipose tissue produce a novel adipo-myokine called metrnl, which is released into circulation. Glial cell differentiation is regulated by metrnl^67^.

Our results, which demonstrated a significant decline in mitochondrial activity in the VPA group, supported these findings. However, training increased the VPA + Training group’s mitochondrial activity, which is linked to the favorable regulation of the growth of brown fat cells with more intact mitochondria. Additionally, metrnl enhances glucose tolerance and energy homeostasis^68^. Metrnl could additionally decrease lipid-induced inflammation using pathways that are dependent on PPARδ or AMPK^69^.

In the current investigation, the VPA group’s serum irisin and cerebellar BDNF levels were considerably lower than those of the control group. VPA administration reduced BDNF mRNA levels in PND 14, especially in the hypothalamus^70^. Conversely, treadmill training increased levels of irisin and BDNF. These findings were consistent with earlier research^20,71^. Training has been suggested to have an impact on autism through mechanisms such as increased BDNF and decreased Bax expression^72^. Irisin is a cerebellar neuropeptide that is also referred to as an exercise-induced myokine. The majority is released by skeletal muscle, with lesser quantities coming from the brain, pancreas, adipose tissue, and heart. Irisin affects brain function via altering neurotransmitter release^73^. According to Kara et al., irisin may contribute to the etiopathogenesis of ASD and could serve as a tracking and diagnostic indicator for ASD^19^**.** Irisin stimulates the synthesis of BDNF in several brain regions which considered as one important neurotrophin that serves as a multipurpose regulator of neural circuit development and maturation. Irisin regulates the expression of BDNF, which may reduce neuronal damage, increase the expression of antioxidant enzymes, decrease proinflammatory cytokines, and stimulate neurogenesis in the brain, especially in the cerebellum^74^. We hypothesized that irisin would mediate the communication between the central nervous system and the muscles during physical activity.

PPAR-γ is a transcription factor widely localized in the CNS^11^. According to our findings, the VPA group had significantly lower levels of PPAR-γ. Previous results indicate that VPA changed the biology of peroxisomes by reducing PPAR-γ signaling and expression^75^**.** Recent investigations have shown that PPAR-γ boosting therapy can effectively preserve neurons and reduce core symptoms in animal models of ASD^76^. By comparing the VPA + Training group to the VPA mice, our data showed a considerable up-regulation of PPAR-γ in the cerebellum, which was consistent with earlier research^77^. By lowering IL-6 and TNF-α and raising GSH, PPAR-γ activation provided neuroprotection against inflammation and oxidative stress^78^ which may contribute to improved neurobehavioral abnormalities in mice.

VPA significantly reduces the expression of cerebellar AMPK as compared to the control group. The down regulation of AMPK/SIRT1/PGC1α signaling and malfunctioning of amygdala parvalbumin interneurons may be brought on by elevated ROS and neuroinflammation with VPA^79^.

However, our study demonstrated that training significantly up regulated the expression of the intracellular energy sensor AMPK. Training may increase mitochondrial biogenesis, which is mediated by AMPK and PPAR-γ activation^80,81^. AMPK activation decreased neuroinflammation by reducing the synthesis of inflammatory mediators and enhancing AMPK/Nrf2 signaling, which had anti-inflammatory qualities^14^**.** Furthermore, AMPK suppresses the NFκB signaling pathway to produce a strong anti-inflammatory impact^82^. Training increased AMPK phosphorylation and BDNF expression, which have an impact on cognitive performance^83^. Metrnl markedly augmented AMPK phosphorylation. These findings shed light on one potential pathway through which metrnl-mediated AMPK activation may be linked to the health benefits of exercise^82^.

## Conclusion

According to our research, VPA caused cerebellar lipid peroxidation by weakening the antioxidant neural defense mechanism. ASD-related behavioral impairment was caused by neuronal excitation with down-regulated metrnl, AMPK, and PPAR-γ in the cerebellum. Low levels of irisin, cerebellar citrate synthetase, and serotonin associated with high levels of inflammatory cytokines may share in ASD pathophysiology. Conversely, Training reduced the social, behavioral, and biochemical signs of autism caused by VPA in mice through antioxidant, anti-inflammatory, and anti-apoptotic mechanisms, with an increase in cerebellar level AMPK, PPAR-γ, and metrnl.

## Data Availability

All data generated or analyzed during this study are included in this published manuscript.
